# Emerging Links Between Ferroptosis and Neurodegeneration: Implications for Disease Mechanisms and Nutraceutical Interventions

**DOI:** 10.1002/fsn3.70385

**Published:** 2025-06-05

**Authors:** Anwar Ali, Quratulain Babar, Ayesha Saeed, Domenico Sergi, Nenad Naumovski, Isam A. Mohamed Ahmed, Ghalia Shamlan, Halah Abdulrahman Hafiz, Muhammad Faisal Manzoor, Joanna Trafialek, Felix Kwashie Madilo

**Affiliations:** ^1^ Institute of Human Nutrition Sciences Warsaw University of Life Sciences–SGGW Warsaw Poland; ^2^ Department of Biochemistry Government College University Faisalabad Pakistan; ^3^ Department of Translational Medicine University of Ferrara Ferrara Italy; ^4^ Discipline of Nutrition and Dietetics, Faculty of Health University of Canberra Canberra Australian Capital Territory Australia; ^5^ Department of Food Sciences and Nutrition College of Food and Agricultural Sciences, King Saud University Riyadh Saudi Arabia; ^6^ Department of Clinical Nutrition, Faculty of Applied Medical Sciences Umm Al‐Qura University Makkah Saudi Arabia; ^7^ Guangdong Provincial Key Laboratory of Intelligent Food Manufacturing School of Food Science and Engineering, Foshan University Foshan China; ^8^ Faculty of Sciences and Technology ILMA University Karachi Pakistan; ^9^ Food Science and Technology Department Ho Technical University Ho Ghana

**Keywords:** aging, ferroptosis, inhibitors, neurodegenerative disorders, nutraceuticals, phytochemicals

## Abstract

A relatively recently identified type of controlled cell death termed ‘ferroptosis’ is driven by iron and characterized by reactive oxygen species (ROS) buildup and lipid peroxidation. This review explores the complex mechanisms underpinning ferroptosis and its potential effects on the onset and development of neurodegenerative diseases. The impact of lipid peroxides, glutathione/glutathione peroxidase 4 (GSH/GPX4), and iron metabolism‐targeting small molecule inhibitors and the involvement of nucleic acids, proteins, and phytochemicals as inhibitors of ferroptosis will be discussed. Additionally, we explore the potential of ferroptosis as a therapeutic target for managing neurodegenerative disorders and address the challenges faced in clinical translation. The emerging research on novel drug delivery approaches and nanomaterial‐based strategies for efficient ferroptosis inhibition is also described. In conclusion, this review provides a detailed overview of the complex landscape of ferroptosis, providing insights into its molecular mechanisms, inhibitors, and potential therapeutic applications. Understanding the multifaceted role of ferroptosis in disease pathogenesis will pave the way for developing innovative interventions to harness its therapeutic potential in various neurological conditions.

## Graphical abstract

Ferroptosis, a controlled cell death, is driven by iron and characterized by reactive oxygen species and lipid peroxidation. The impact of lipid peroxides, GSH/GPX4, and iron metabolism–targeting small molecule inhibitors is discussed. Nucleic acids, proteins, and phytochemicals are implicated as inhibitors of ferroptosis.

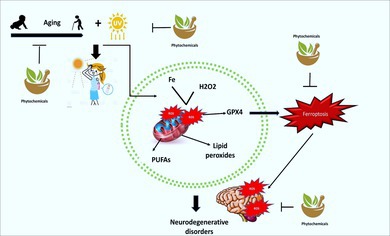

## 1 Introduction

Over the past decades, several models have been proposed to elucidate the underlying biological mechanisms of aging (Dice 1993). Among these models, the *‘oxidative stress hypothesis’* has emerged as a prominent theory, building upon the foundational free radical concept of aging (Ghezzi et al. 2017). The emerging research has suggested intriguing links between ferroptosis, a regulated form of cell death driven by iron‐dependent lipid peroxidation, and neurodegenerative diseases (Yan et al. 2021). The association between ferroptosis and neurodegeneration was proposed as potential implications for disease mechanisms and interventions in aging‐related disorders (Coradduzza et al. 2023). There is a growing body of evidence linking ferroptosis to neurodegeneration. This hypothesis suggests that oxidative damage arises from uncontrolled reactive oxygen species generation, the combination of these factors, and a decrease in the activity/expression of detoxifying enzymes, which play a crucial role in regulating oxidative stress levels (Buffenstein et al. 2008; Pérez et al. 2009).

Oxidative stress may arise due to an imbalance between the production of pro‐oxidant species and molecules with antioxidant capacity, along with the activity of detoxifying enzymatic systems. This imbalance can further lead to cellular and molecular damage, as reactive oxygen species and other reactive species inhibit the cellular defense mechanisms, causing oxidative damage to biomolecules (Conti et al. 2016), including DNA, proteins, and lipids, resulting in cell death and impacting the longevity and function of organ systems (Dai et al. 2014; Scheibye‐Knudsen et al. 2015). While reactive oxygen species, including hydrogen peroxide, hydroxyl radical, peroxynitrite, superoxide, reactive nitric oxide, and reactive lipid aldehydes, are constantly generated and thought to contribute to age‐related redox instability, their effects are balanced by the presence of a highly efficient cellular antioxidant system. These systems comprise antioxidant enzymes and molecules with antioxidant capacity (Chung et al. 2009; Lennicke et al. 2015). The term ‘inflammaging’ is commonly ascribed to the chronic low‐grade inflammation that occurs as individuals age, and it has been proposed as a significant factor linking physiological changes during aging to the development of age‐related degenerative illnesses (Franceschi et al. 2018).

**FIGURE 1:**
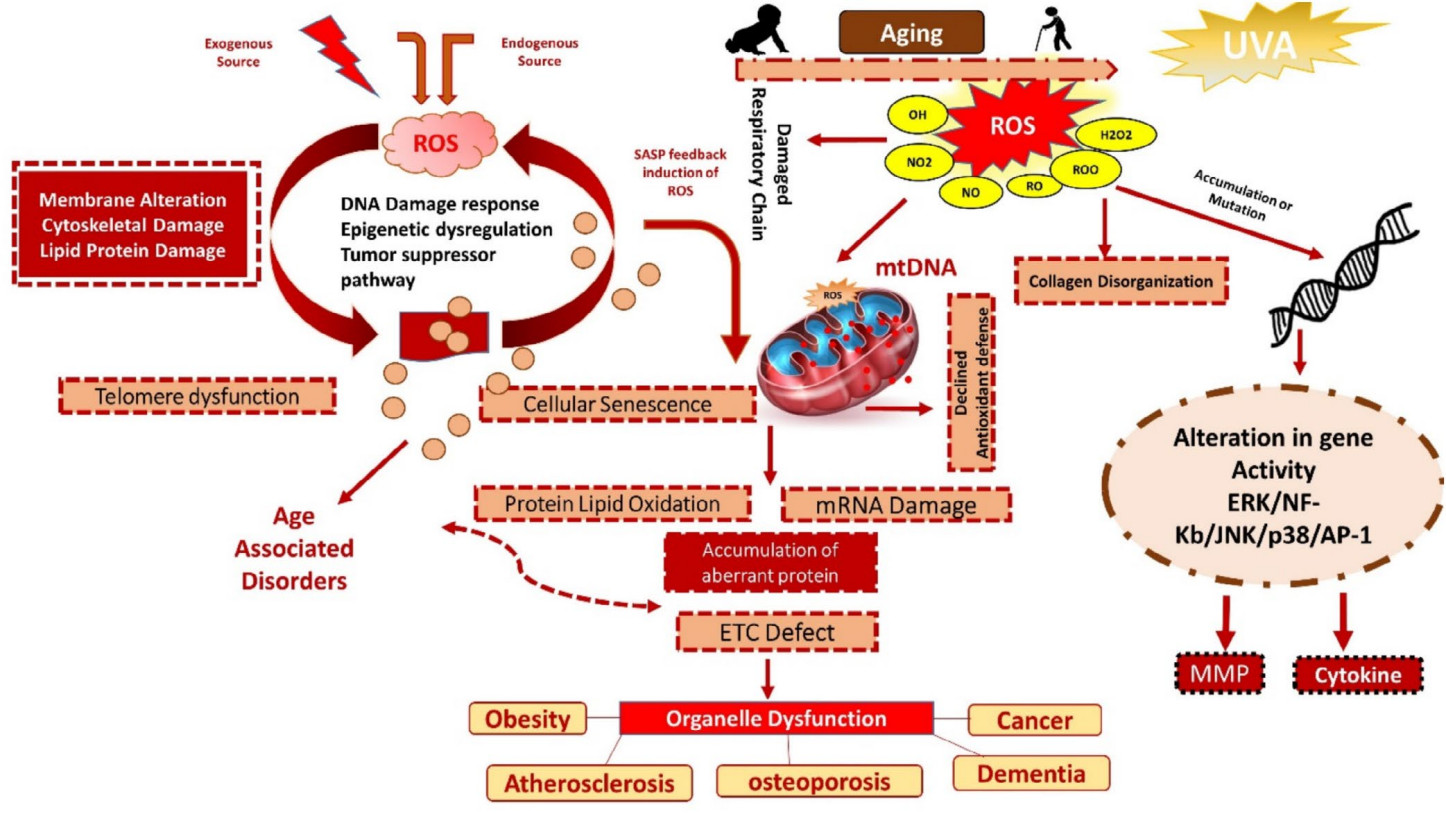
Aging leads to altered cellular functioning, followed by oxidative stress, which causes age‐related disorders.

Additionally, inflammation is tightly linked with oxidative stress, with this relationship being termed oxinflammation (Valacchi et al. 2018). Indeed, while reactive oxygen species and other reactive molecules can perpetuate inflammatory responses (Andrade et al. 2022), inflammation can also fuel oxidative stress. It is proposed that foods high in antioxidants may reduce the effects of oxidative stress and the risk of developing several noncommunicable diseases (Tan et al. 2018). Consequently, the antioxidant activity of plant‐based products has generated significant interest among industrial and academic experts in their attempt to manage the negative consequences of age‐related disorders (Figure 1).

While iron is essential for the proper functioning of several metabolic pathways, it is involved in oxygen transport. Dysregulated iron metabolism can lead to its hazardous accumulation as a redox center in the electron transport chain, particularly in the brain (Singh et al. 2019). An iron‐mediated buildup of lipid peroxides induces a type of programmed cell death known as ferroptosis (Su et al. 2019). This novel concept bridges the gap between iron homeostasis and lipid peroxidation, introducing a critical mechanism underlying the process of ferroptosis. Iron is a potent pro‐oxidant that can participate in Fenton‐like reactions, forming highly reactive lipid peroxides that may trigger ferroptotic cell death (Kajarabille and Latunde‐Dada 2019; Timoshnikov et al. 2022). The disruptions in iron homeostasis can result in an excessive free iron buildup, promoting ROS formation through Fenton reactions (Wojtunik‐Kulesza et al. 2019). Three primary mechanisms that may contribute to ferroptosis include the oxidation of membrane polyunsaturated fatty acids (PUFAs), glutathione degradation via the glutathione peroxidase‐4 pathway (GPX4), and the intracellular accumulation of free iron (Cao et al. 2016; Tang and Kroemer 2020).

The onset and propagation of ferroptosis involves a complex interplay of various metabolic processes, including forming lipid peroxides. These reactive lipid species contribute to cellular membrane damage and subsequent cell death (Kagan et al. 2017; Ploumi et al. 2022). The role of GPX4, a critical antioxidant enzyme that suppresses lipid peroxidation by reducing lipid hydroperoxides, has been extensively studied within the context of ferroptosis regulation (Krümmel et al. 2021; Xu, Sun, et al. 2021). Inhibition of GPX4 activity has been associated with increased lipid peroxidation and enhanced susceptibility to ferroptotic cell death (Forcina and Dixon 2019; Yang and Stockwell 2016). Understanding the intricate metabolic processes involved in ferroptosis and the significance of lipid peroxide formation provides promising implications for developing targeted therapeutic interventions to modulate this unique form of cell death (Li, Cao, et al. 2020). Several pathways are involved in ferroptosis regulation, including iron transport and storage mechanisms, lipid peroxidation, and the antioxidant defense system (Sahoo and Sharma 2023; Su et al. 2019). Cellular uptake of iron via transferrin receptor 1 (TFR1) and its release from ferritin‐controlled iron stores play crucial roles in maintaining iron homeostasis (Mounsey and Teismann 2012; Zhang et al. 2022).

This comprehensive review explores the relationship between iron and lipid metabolism and the intricate network of associated processes underlying ferroptosis. Furthermore, we aim to discuss potential therapeutic strategies that target ferroptosis pathways, paving the way for novel interventions in diseases where dysregulated iron and oxidative stress play significant roles.

## 2 Ferroptosis in Neurodegenerative Disease

Each neurodegenerative condition has an early preclinical period during which symptoms are nonexistent or mild, and the lesion is limited to one or a small number of brain regions, only affecting the most vulnerable cells and microcircuits (Mitoma et al. 2020). In these diseases, protein aggregation in specific cell types causes a localized onset followed by neuronal dropout. The pattern of regional injury appears to reflect a network‐based environment, contradicting the idea that disease spreads over the cortical mantle through spatial contiguity, according to postmortem and in vivo neuroimaging studies. The spread happens either as unidirectional movements or as oligofocal processes when few regions experience it simultaneously, and in this case, the axonal connections of the most sensitive cells within the onset region ultimately determine the later‐affected regions (Seeley 2017). Without connectional spread, the symptoms would emerge in different locations at different times. In this instance, anatomical progression depicts discrete, temporally spaced disease eruptions throughout numerous (but not necessarily linked) locations. The advancement process exists independently from connectivity because it depends on cellular and regional sensitivities to pathogenic factors that spread widely throughout the body. In this sense, advancement is unrelated to connectivity and is brought about by a gradated hierarchy of cellular and/or regional susceptibilities to a pathogenic process that is widely expressed (Seeley 2017).

Iron is utilized in the cellular metabolism of the Central Nervous System (CNS) for ATP production through its involvement in mitochondrial electron transport proteins. These proteins play a crucial role in oxidative phosphorylation, transferring electrons along the mitochondrial electron transport chain, ultimately leading to ATP synthesis. However, using iron in the CNS's cellular metabolism can also make it susceptible to oxidative damage when there is an excess of iron and a depletion of antioxidant defense mechanisms (Urrutia et al. 2021). Ferroptosis is characterized by a cascade of events that involve lipid peroxidation, glutathione depletion, and the synchronous buildup of iron in the brain (Bertrand 2017). The consequences of these events may lead to astrocyte dysregulations, myelin sheath degradation, impaired neurotransmission, cell death, and the development of dementia (Brandebura et al. 2023).

**FIGURE 2:**
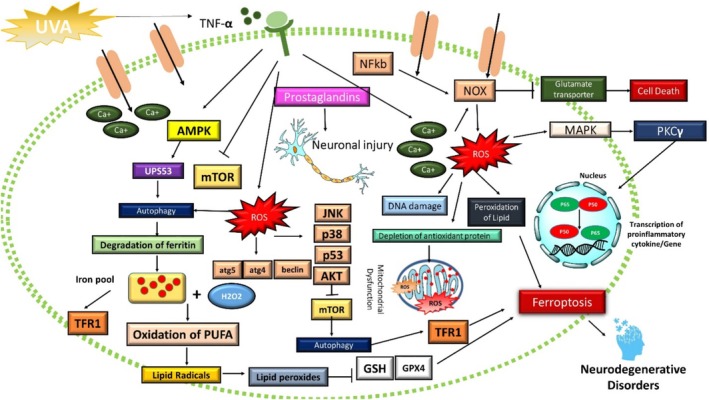
Cellular signaling of ferroptosis followed by lipid peroxidation, apoptosis, and altered cellular oxidative stress.

Detecting ferroptosis in vivo is challenging due to a lack of relevant biomarkers. However, extensive evidence suggests that ferroptosis contributes to the pathogenesis of neurodegeneration (David et al. 2022; Reichert et al. 2020; Ryan et al. 2023; Vitalakumar et al. 2021). Additionally, reduced expression of the glutathione system and GPX4 has been linked with ferroptosis in motor dementia (DeGregorio‐Rocasolano et al. 2019; Dias et al. 2013; Gu et al. 2015; Johnson et al. 2012). Given the role of ferroptosis in promoting neurodegeneration and the significance of the antioxidants in counteracting this process, inhibiting ferroptosis while enhancing GPX4 could be a potential therapeutic strategy for addressing neurodegeneration (Figure 2) (Cardoso et al. 2017).

### 2.1 Alzheimer's Disease (AD)

Alzheimer's disease (AD) is an irreversible, multifactorial neurodegenerative disease characterized by memory loss and cognitive impairment. From the pathological perspective, AD is underlined with severe neuronal apoptosis, excessive accumulation of extracellular amyloid‐β (Aβ) between the neurons, abnormal hyperphosphorylation of tau protein that forms intraneuronal neurofibrillary tangles, and reduction in cerebral glucose metabolism (D'Cunha et al. 2019).

Synapse loss and oxidation of neurotransmitters are also prevalent in AD and may lead to accelerated cognitive impairment that is potentially initiated by increased chronic oxidative stress (Butterfield and Boyd‐Kimball 2020; Markesbery and Carney 1999; Querfurth and LaFerla 2010). The iron imbalance was also associated with accelerated neurodegeneration and generation of ROS in AD (Querfurth and LaFerla 2010). Increased free iron availability initiates and maintains Tau phosphorylation, a process implicated in the abnormal modification of Tau protein, and is associated with neurofibrillary tangles, characteristic of tauopathies, a group of neurodegenerative disorders (J Bonda et al. 2011; Reichert et al. 2020; Serrano‐Pozo et al. 2011). Additionally, oxidative stress interferes with Aβ and tau proteins, contributing to their dysfunction and aggregation (Pohanka 2014).

Amyloid precursor protein (APP) and tau proteins work synergistically to facilitate iron transfer, and dysfunctions reported in these proteins have been implicated in iron accumulation (Wu et al. 2018). Furthermore, iron deposition due to these protein dysfunctions leads to increased ROS generation in the cerebrospinal fluid brains of individuals with AD (Ayton, Faux, et al. 2015).

In animal models of AD, the down‐regulation of the guanine‐rich RNA sequence binding factor 1 (GRSF1), which controls glutathione peroxidase 4 (GPX4) translation, has been observed (Tuo et al. 2017). The GPX4 knockdown in mice results in age‐dependent neurodegenerative changes and substantial neuronal loss, exacerbated by a diet low in vitamin E (Hambright et al. 2017). The GPX4 and vitamin E are both naturally occurring inhibitors of ferroptosis. The ablation of GPX4 and glutathione depletion (GSH) worsen cognitive impairment and neurodegeneration in animal models, supporting the connection between ferroptosis and AD pathogenesis (Ghosh et al. 2014; Hambright et al. 2017). Selenium (Se), a crucial regulator of GPX4 function and essential for brain health, is associated with cognitive impairment and AD pathology. However, it is essential to be cautious about excessive dosages of selenium, as they may lead to neurotoxicity (Chmatalova et al. 2017; Vinceti et al. 2017).

In studies conducted on AD mice, modulation of ferroptosis has been explored as a potential therapeutic approach. For instance, the use of α‐lipoic acid has been found to inhibit P38 and maintain GPX4 activity, thereby counteracting neurotoxicity and cognitive dysfunction associated with ferroptosis. By maintaining GPX4 activity, α‐lipoic acid exhibits a specific link to ferroptosis‐related pathways, contributing to its neuroprotective effects (Liu et al. 2020; Wu et al. 2018).

### 2.2 Parkinson's Disease (PD)

A recognizable clinical syndrome with a variety of etiologies and clinical manifestations is Parkinson's disease (PD) (Bloem et al. 2021). It is not caused by an infection, which means microbial pathogens can contribute to developing the environment but are not directly involved in causing PD. However, it's becoming common and spreading rapidly around the world. According to the Parkinson's Foundation, 10 million people are living with PD worldwide. The projected PD rate from 2021 to 2050 is 196% (Su et al. 2025). Ninety genetic risk variations together account for 16%–36% of the heritable risk of non‐monogenic Parkinson's disease, while 3%–5% of Parkinson's disease in most groups is explained by genetic reasons connected to known Parkinson's disease genes, or monogenic Parkinson's disease (Bandres‐Ciga et al. 2020). Constipation, not smoking, and having a family with Parkinson's disease or tremor are other causative correlations, each of which at least doubles the chance of Parkinson's disease (Bloem et al. 2021).

The gradual deposition of free iron is one of the characteristics of Parkinson's disease (PD), along with the diminution of striatal dopamine, absence of neuromelanin, and presence of intracellular Lewy bodies with accumulated α‐synuclein as the core component. Furthermore, the progressive degeneration of dopaminergic neurons in the substantia nigra's pars compacta (SNpc) is a brain region critical for motor control and coordination (Dauer and Przedborski 2003; Lees et al. 2009). As PD progresses, a decrease in antioxidant enzymes within the glutathione system leads to mitochondrial dysfunction and lipid peroxidation. These interconnected factors contribute to neuronal death and impair the adequate functioning of the nervous system (Dorszewska et al. 2021).

The association between iron accumulation and PD is typically characterized by increased iron levels in specific brain regions, particularly the substantia nigra pars compacta and the basal ganglia (Dexter et al. 1992; Gorell et al. 1997). In addition, mutations in various genes involved in brain iron homeostasis (iron response elements (IRE) in their 5′‐and 3′‐UTR mRNA) have been identified in PD patients, further supporting the involvement of iron metabolism in the disease (Rhodes and Ritz 2008).

While not involving apoptosis, the 1‐Methyl‐4‐phenylpyridinium (MPP (+)) treated SH‐SY5Y cell line exhibits specific characteristics shared with ferroptosis, including lipid peroxidation, which 3,3′‐diindolylmethane (DIM) and ferostatin‐1 (Fer‐1) can prevent. Iron chelators have been found to suppress ferroptosis and protect dopaminergic neurons from cell death (Ayton and Lei 2014). Additionally, in a mouse model induced by 1‐methyl‐4‐phenyl‐1,2,3,6‐tetrahydropyridine (MPTP), a neurotoxin commonly used to mimic Parkinson's disease pathology in mice, GSH depletion suggested potential associations with ferroptosis, although a direct causal link has not been definitively established (Feng et al. 2014; Wüllner et al. 1996).

GSH depletion and iron accumulation co‐occur, which causes the —OH radicals and lipid peroxidation in ferroptosis to develop (Guiney et al. 2017). Consequently, the accumulation of lipid peroxides damages cell membranes and organelles, ultimately resulting in cell death. Moreover, the relationship between GSH and iron extends beyond lipid peroxidation. GSH is a critical regulator of iron homeostasis by binding to ferrous iron in the labile iron pool (LIP), preventing oxidation, and maintaining iron in a soluble, *‘non‐toxic’* form (Guiney et al. 2017; Hider and Kong 2011). By inhibiting the catalytic function of ferrous iron in synthesizing highly reactive hydroxyl radicals (—OH) from biologically accessible hydrogen peroxide, GSH plays a vital role in limiting oxidative stress and protecting cells from iron‐induced damage (Valko et al. 2016).

In Vitro, Studies have shown that the administration of erastin, which disrupts the xCT cystine/glutamate antiporter and reduces cystine absorption, leads to ferroptotic cell death (Dixon et al. 2012). The decreased cysteine absorption caused by erastin reduces GSH production, as cystine is a precursor of GSH synthesis (Bannai 1986). Consequently, the cystine/glutamate antiporter disruption triggers ferroptosis through GSH depletion and iron‐induced oxidative damage (Cong et al. 2022).

Overall, the complex interplay between GSH, iron, and lipid peroxidation in the context of ferroptosis highlights the importance of GSH as a key regulator of cell survival and iron homeostasis (Rochette et al. 2022). Understanding these intricate mechanisms provides valuable insights into the pathogenesis of ferroptosis and its potential implications for various diseases, including neurodegenerative disorders like Parkinson's disease and Alzheimer's disease.

### 2.3 Huntington's Disease (HD)

Patients and their families suffer significantly from Huntington's disease (HD), which has a progressive trajectory, onset usually in the prime of adulthood, autosomal dominant heredity, and a mix of motor, cognitive, and behavioral symptoms (Bates et al. 2015). The enlarged CAG trinucleotide repeat (of varying length) in the HTT gene, which codes for the huntingtin protein, is the cause of the illness. Abnormally long polyglutamine sequences found in carriers of mutations cause huntingtin to be created, which can lead to hazardous gains in function and the protein's fragmentation, which can cause neuronal malfunction and death (Tabrizi et al. 2020).

HD progression is also partially associated with ferroptosis. Although iron accumulates in neurons in HD animal models compared to wild‐type mice, it is essential to note that ferroptosis in HD is not solely dependent on iron accumulation (Chen et al. 2013). The iron buildup in neurons raises the possibility that iron may play a key pathogenic role in HD (Rosas et al. 2012). Furthermore, the mutant huntingtin protein in HD leads to elevated levels of ROS and enhanced oxidative stress within cells (Wyttenbach et al. 2002). Further investigation is needed to understand the interplay between ferroptosis and HD pathogenesis, considering other potential mechanisms and cell types affected by the mutant htt protein.

Glutathione (GSH) is reported to regulate the expression of GPX4, suppressing ferroptosis and eliminating excess lipid peroxides (Mi et al. 2019). However, ROS production increases in HD, leading to elevated lipid peroxides and GSH depletion, thereby reducing GPX4 levels and promoting ferroptosis (Reichert et al. 2020). The dysregulation of GSH in HD patients affects the promotion of ferroptosis and impacts the functioning of enzymes that rely on GSH for their activity (DiFiglia et al. 1995; Mi et al. 2019). This dysregulation can have broader consequences on cellular processes and contribute to the pathogenesis of HD. The interplay between GSH, ROS, GPX4, and ferroptosis highlights the complexity of the disease's mechanisms and offers potential targets for therapeutic interventions.

## 3 Plant‐Based Neuroprotective Phytochemicals

**FIGURE 3:**
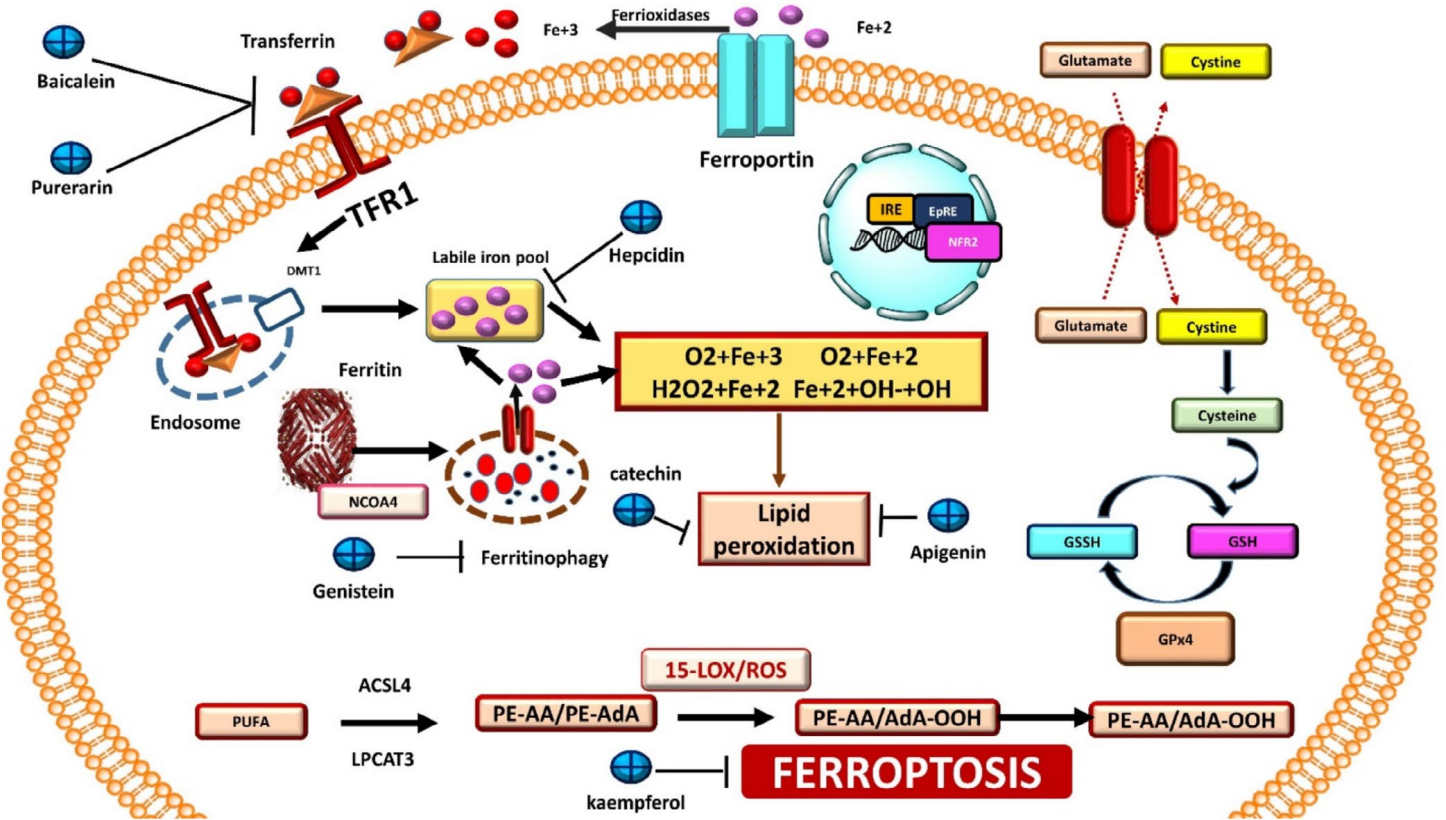
Inhibition of ferroptosis by plant‐based phytochemicals.

Neuroprotection by foods and bioactives has been suggested to mitigate the onset and progression of neurodegenerative disorders such as PD and AD (Babazadeh et al. 2023; Naoi et al. 2019). Plant phytochemicals can protect neurons by targeting various pathogenic factors, including apoptosis, aberrant protein accumulation, neurotrophic factor deficits, oxidative stress, and mitochondrial dysfunction (Fisette et al. 2022; Naoi et al. 2019). Neuroprotection is a *‘disease‐modifying’* therapy that aims to improve brain function, repair neural networks, and prevent neuronal death in conditions such as AD, depression, and PD (Figure 3) (Schapira and Olanow 2004). Complex molecular interactions underlie the potential of some nutraceuticals to prevent ferroptosis. Polyphenolic compounds such as resveratrol exhibit strong antioxidant properties through their ability to modify glutathione and lipid metabolism pathways (Lesjak et al. 2022). By reducing lipid peroxidation, this process stops ferroptosis (Babar et al. 2023). Turmeric's derivative curcumin has various benefits, one of which is the upregulation of the production of the antioxidant enzyme GPX4, which is essential in reducing the death of cells caused by lipid peroxidation (Stepanić and Kučerová‐Chlupáčová 2023). Green tea (EGCG) and other fruit polyphenols effectively prevent ferroptosis by controlling iron metabolism, lipid peroxidation pathways, and antioxidant cascades (Li et al. 2023). The area of treating neurodegenerative diseases is actively researching their neuroprotective characteristics. Though these nutraceuticals exhibit promising properties in inhibiting ferroptosis via antioxidative and anti‐inflammatory pathways. Scientists within neurodegenerative disease therapy seek to understand neuroprotective capabilities regarding nutraceuticals that demonstrate potential ferroptosis‐blocking properties through antioxidative and anti‐inflammatory properties together with diverse neuroprotective benefits. Further clinical research is necessary to determine the precise molecular targets, determine the most effective dosages, and verify the clinical utility of these nutraceuticals in treating neurodegenerative disorders (Sun et al. 2022). Rich in fruits and vegetables, flavonoids like luteolin and quercetin show potential in reducing ferroptosis by adjusting lipid peroxidation pathways and enhancing antioxidant defenses within cells. These substances' antioxidative and anti‐inflammatory qualities have drawn attention to them as prospective treatments for neurodegenerative diseases.

Some of the nutraceuticals such as epigallocatechin‐3‐gallate (EGCG), hesperidin, hesperetin‐rutinoside, huperzine, luteolin, myricitrin, proanthocyanidins, and quercetin have been shown to enhance electron transport chain (ETC) activity (Forbes‐Hernández et al. 2014; Valenti et al. 2013). Nutraceuticals promote mitochondrial biosynthesis and contribute to neuronal protection (de Oliveira et al. 2016). The neuroprotective mitochondrial effects play an essential role, but scientists have not yet proven their direct involvement with anti‐ferroptosis. Research needs to clarify which effects of these compounds act directly in ferroptosis.

The primary way that bioactive substances and nutraceuticals such as resveratrol, curcumin, flavonoids, and polyphenols affect mitochondrial biogenesis is by activating PGC1alpha, which is a critical regulator of mitochondrial activity (Chuang et al. 2019). By increasing PGC1alpha activity, these substances improve mitochondrial biogenesis, or the process of making new mitochondria. By maximizing fusion and fission events, this activation affects mitochondrial dynamics and enhances mitochondrial efficiency and cellular energy generation (de Oliveira et al. 2016). This interplay highlights the potential of these chemicals in increasing mitochondrial vitality and cellular resilience against various neurodegenerative diseases, hence supporting cell health and function. Research shows these effects could benefit patients with neurodegenerative diseases, though their relationship to ferroptosis needs further explanation. Relatively recent research indicates that nutraceuticals have the potential to regulate mitochondrial function by controlling the formation of the mitochondrial permeability transition pore (mPTP), influencing pore opening in the outer mitochondrial membrane, and blocking the efflux of calcium and cytochrome c, thus preventing cell death through apoptosis (Naoi et al. 2019). Flavonoids modulate mitochondrial apoptosis pathways, regulating cell survival (Ref). Neurotrophic factors (NTFs) such as GDNF and BDNF are crucial for maintaining neuronal health, and their deficiencies are linked to psychiatric disorders, PD, and AD (Zhang et al. 2006). GDNF and BDNF have shown some success as potential targeted therapies in neurodegeneration, with positive outcomes reported in PD patients treated with GDNF infusion (Hegarty et al. 2017). The relationship between these neurotrophic factors and ferroptosis in terms of their direct impact remains unknown. The selection of nutraceuticals, such as resveratrol, curcumin, and flavonoids, was reported to have neurotrophic‐like actions predominantly due to their high lipophilicity and can potentially offer neuroprotection (Youdim et al. 2004).

Polyphenols activate pro‐survival signaling pathways such as MAPK mechanisms (PKC and PI3K/Akt), influencing neuroplasticity, memory, and cellular function (Uddin et al. 2020). Flavonoids, including resveratrol, quercetin, and luteolin, were reported to protect against neuronal cell death and reduce oxidative stress‐induced apoptosis (Naoi et al. 2017). The research about polyphenolic compounds' protective effects on ferroptosis requires additional investigation to establish their complete relationship. Polyphenols also enhance neurotrophic factors like GDNF and BDNF, improving neurobiology. The research about polyphenolic compounds' protective effects on ferroptosis requires additional investigation to establish their complete relationship. Phytochemicals like astaxanthin, curcumin, and resveratrol boost BDNF levels in neurodegenerative models (Fanaei et al. 2016; Singh et al. 2011).

In neurodegenerative diseases such as AD and PD, abnormal protein accumulations such as senile plaques, neurofibrillary tangles, neurites, and Lewy bodies are common. Nutraceuticals have shown promise in targeting protein aggregation, reducing fibrils and oligomers, and promoting the clearance of amyloid beta (Aβ) aggregates, offering potential neuroprotective effects against these disorders (Bieschke 2013; Smid et al. 2012). Research needs to explore how the neuroprotective attributes of the mentioned effects contribute to anti‐ferroptosis mechanisms. See Table 1 for a summary of nutraceuticals with protective effects against neurodegenerative diseases.

**TABLE 1:** Neuroprotective mechanisms of nutraceuticals.

Sr. no.	Nutraceuticals	Mechanism of action	References
1	(7,8‐DHF), (7,8,3‐THF), fisetin, deoxygedunin	Activates PI3K‐Akt, Ras‐MEK (MAPK)‐ERK pathways, (CREB)	Liu et al. (2016)
2	Alcohol derivatives of ferulic acid and Aldehyde	Prevents the mitochondrial permeability transition pore (mPTP) from forming by suppressing the pore opening	Naoi et al. (2019)
3	Altenusin	Inhibits tau hyperphosphorylation	Chua et al. (2017, Park et al. (2008)
4	Amentoflavone	Activates PI3K/Akt and PKC protects neurons from cell death	Hwang et al. (2009)
5.	Anthocyanins	Protects complex I, modulated mitochondrial fission/fusion pathways	Parrado‐Fernández et al. (2016)
6	Anthraquinones	Inhibits tau aggregation	Pickhardt et al. (2005)
7	Astaxanthin	Increases BDNF	Leem et al. (2014), Xu et al. (2010)
8	Astaxanthin and black tea extract	Prevents membrane permeabilization and protects cells from apoptosis	Camilleri et al. (2013)
9	Caffeic acid	Activates ERK‐CREB‐BDNF or PI3K/Akt‐ cascade	Zhang et al. (2015)
10	Carnosic acid, carnosol and hydroxytyrosol	Induces translocation of phosphorylated Nrf2, enhances glutathione reductase, glutathione peroxidase, S‐transferases, superoxide dismutase, and NQO1	Qin et al. (2014)
11	Catalpol	Induction of GDNF	Naoi et al. (2017)
12	Compounds of sesamolin and ferulic acid that are lipophilic	Prevents the inner mitochondrial membrane's cyclophilin‐D and adenine nucleotide translocator (ANT) from forming a pore	Naoi et al. (2019)
13	Curcumin	Prevents the important thiol residues from being oxidized and prevents the voltage‐dependent anion channel from being phosphorylated	Naoi et al. (2019)
14	Deoxygedunin	Increases TrkB and BDNF expression	Jang et al. (2010), Li et al. (2013)
15	Derivatives of quinic acid and spicoside A	Increases neurite outgrowth and neuroprotective potential when binding to the TrkA receptor	Hwang et al. (2011)
16	EGb 761, ginkgolide	Inhibits MAO	Carradori et al. (2016)
17	EGCG	Potentiates neuroprotective and neurogenic action	Umeda et al. (2008)
18	EGCG	Regulates PKCα and PKCε, increased BDNF, TrkA, and TrkB expression	Kalfon et al. (2007), Liu et al. (2014), Menard et al. (2013)
19	Epicatechin	Inhibits (p75NTR) prevent retinal neurodegeneration.	Al‐Gayyar et al. (2011)
20	Flavonoids	suppresses Jun N‐terminal kinase activation, stimulates upstream MAPK‐kinase‐kinase, and inhibits oxidative stress‐induced apoptosis	Hwang et al. (2009)
21	Genistein	Induces mitochondrial permeability transition and oxidizes thiol and pyridine nucleotide	De Marchi et al. (2009)
22	Genistein and galangin	Improves behavioral impairment	Mori et al. (2013), Mori et al. (2012)
23	*Ginkgo biloba*	Enhances serum BDNF	Sadowska‐Krępa et al. (2017)
24	Ginkgolide B	Inhibits MAO‐A and induced BDNF	Wang et al. (2017)
25	Green tea catechin	Increases plasma BDNF levels	Neshatdoust et al. (2016), Singh et al. (2011)
26	Hesperetin	Increases GDNF and BDNF expression, promoted cell survival	Xu et al. (2013)
27	Hesperetin	Activates Akt, down‐regulate ASK1), Bad, caspase‐9	Vauzour et al. (2007)
28	Honokiol	Inhibits Aβ aggregation	Das, Stark, et al. (2016)
29	Icariin, rutin	Reduces the expression of β‐secretase	Kostomoiri et al. (2013)
30	Kaempferol	Inhibits αSyn oligomer formation	Pickhardt et al. (2005), Takahashi et al. (2015)
31	Kaempferol, baicalein, wogonin	It opens the mPTP, inhibits Bcl‐2 and Bcl‐xL, induces the oligomerization of the Bax protein, and down‐regulates the NF‐B signal pathway	Gorlach et al. (2015)
32	Kaempferol, bailarein, and naringenin	Induces antiapoptotic Bcl‐2 and Bcl‐xL, suppresses apoptogenic Bax and Bak, and modulates apoptotic pathways	Naoi et al. (2019)
33	Liquiritigenin	Induces mitochondrial fusion and inhibits mitochondrial fragmentation and cytotoxicity	Basso et al. (2016)
34	Luteolin	Improves ETC activity	Valenti et al. (2013)
35	Nonflavonoid polyphenols	Activates NGF‐specific TrkA receptor	Hwang et al. (2011)
36	Olive polyphenols	Increases TrkB and TrkA	De Nicoló et al. (2013)
37	Polyphenols	Activates PKC	J. Das, Ramani, and Suraju (2016)
38	Polyphenols	Keep1/Nrf2/antioxidant response element (ARE) pathway activation	Qin et al. (2014)
39	Puerarin	Shows NTF‐mimic activity, induces NTF expression	Youdim et al. (2004)
40	Quercetin and wogonin	Induces mitochondrial biogenesis	Nieman et al. (2010)
41	Resveratrol	Improves expression of Opa1, Mfn1, Mfn2, Drp1, and Fis1, protects PC12 cells	Peng et al. (2016), Sandoval‐Acuna et al. (2014)
42	Resveratrol	Inactivation of GSK‐3β by phosphorylated Akt	Zeng et al. (2017)
43	Rosmarinic acid	Decreases the toxic oligomers and fibrils	Ono et al. (2012), Thapa et al. (2016)
44	Selegiline	Activation of neuroprotective signaling pathways	Carradori et al. (2016)
45	Tannic acid	Inhibits β‐secretase	Mori et al. (2012)

## 4 Ferroptosis Inhibitors: Targeting Cell Death Pathways

The field of ferroptosis inhibitors remains relatively limited, with only a few small molecule compounds identified as potential candidates. However, the applications of ferroptosis inhibitors extend beyond. Iron chelators and ferroptosis inhibitors exist as distinct pharmacological agents. Only deferiprone (DFP) has advanced to clinical trials, although other iron‐chelating medications, including VK‐28, M30, and deferoxamine, have shown inhibitory effects on ferroptosis in vitro studies or animal models. The primary function of iron chelators, including deferiprone, deferasirox, and deferoxamine, relies on iron level reduction in the body or cells, while their ferroptosis prevention works as a derivative effect of this primary mechanism. It may be able to cross the blood–brain barrier for this reason (Bar‐Am et al. 2015). A study has demonstrated the therapeutic potential of ferroptosis inhibitors in preventing peroxidation‐related damage in Parkinson's disease model animals (Park et al. 2004). Enhancing GSH production has been suggested with decreased neuronal degeneration in mice models of PD (REF), while early treatment investigations in PD patients have shown modest improvements in motor function (Ayton, Lei, et al. 2015; Monti et al. 2016). Iron‐chelating drugs have also demonstrated the ability to improve motor symptoms in various animal models of PD (Skouta et al. 2014). Inhibitors of ferroptosis have an anti‐inflammatory effect on spinal cord contusion (SCI) by preventing the development of inflammatory factors such as TNF‐α, ICAM‐1, and IL‐1 (Zhang et al. 2019). In treating spontaneous brain hemorrhage, ferroptosis inhibitors like ferrostatin‐1 (Fer‐1) hold promise for preventing secondary brain injury (Zhang et al. 2018). Ferroptosis inhibitors, including DFP, have also shown anti‐inflammatory properties in the treatment of neurodegenerative diseases such as motor neuron disease, AD, and PD, with ongoing investigations into the potential for chelation of iron in treating amyotrophic lateral sclerosis (Devos et al. 2019; Masaldan et al. 2019).

After reperfusion and ischemia, both mouse cortical nerve cells and hippocampal neurons in an in vitro stroke model have displayed evidence of ferroptosis. Furthermore, the application of ferroptosis inhibitors successfully prevented cell death in these models (Choi et al. 2012; Tuo et al. 2017).

Recent research has shed light on the significance of ferroptosis in the development and progression of several disorders, including nervous system malignancies and neurodegenerative diseases (Jiang et al. 2021). The manipulation of ferroptosis through inducers or inhibitors offers novel therapeutic strategies for different conditions. Ferroptosis inducers and inhibitors encompass a range of substances identified to either trigger or prevent ferroptosis (Tang and Kroemer 2020). The pharmacological agents, including erastin, sorafenib, and sulfasalazine, have not received direct studies on their effects on neurodegenerative diseases or the central nervous system (CNS). Future research needs to establish the CNS effects of these pharmacological agents since their brain‐related impacts remain uncertain.

In BJeLR human foreskin fibroblasts, Calu‐1 human lung cancer cells, and HT‐1080 human fibrosarcoma cells, erastin can directly block System Xc function and change glutathione (GSH) synthesis (Dixon et al. 2012). Sorafenib, a clinically used medication for advanced tumors, including thyroid cancer, hepatocellular carcinoma, and renal cell carcinoma, has been found to induce ferroptosis in HT‐1080 cells within a specific dosage range (Dixon et al. 2014). It has been demonstrated that the anti‐inflammatory drug for rheumatoid arthritis, sulfasalazine (SAS), inhibits System Xc and considerably slows the development of lymphoma cells (Gout et al. 2001). Hexamethylmelamine (altretamine), an anticancer medication used for managing ovarian cancer, has been found to impede the repair of lipids by GPX4, suggesting a potential mechanism of action (Woo et al. 2015). Cisplatin, a platinum‐based chemotherapeutic agent, exhibits a high affinity for thiol‐rich biomolecules and can directly bind to GSH, depleting GSH levels and inactivating GPX4. Combining cisplatin with erastin has shown synergistic antitumor activity in human lung cancer cell line A549 and human colon cancer cell line HCT116 (Guo et al. 2018). The introduction of additional ferroptosis inducers or STAT3 inhibitors has been demonstrated to increase the susceptibility of osteosarcoma cells to cisplatin, offering a potential approach to treating drug‐resistant osteosarcoma (Liu and Wang 2019). By encouraging the accumulation of intracellular iron ions, the tyrosine kinase inhibitor lapatinib and the lysosome disruptor siramesine cause ferroptosis in breast cancer cells, indicating a potential treatment method for the disease (Ma et al. 2016).

Regarding ferroptosis inhibitors (Table 2), baicalein suppresses GPX4 degradation, lipid peroxidation, and GSH depletion in pancreatic cancer cells subjected to elastin‐induced ferroptosis. Baicalein has also demonstrated the potential to improve prognosis and recovery in cerebral cortical impact and reduce phosphatidylethanolamine oxidation (Kenny et al. 2019). Rosiglitazone, pioglitazone, and troglitazone have been identified as inhibitors of ACSL4, protecting Pfa1 cells from RSL3‐induced ferroptosis and preventing lipid oxidation. Zileuton, an oral 5‐LOX selective inhibitor used for maintenance therapy in asthma patients, can prevent elastin‐induced ferroptosis in LNCaP and K562 cells that overexpress ACSL4 by inhibiting the synthesis of 5‐hydroxyeicosatetraenoic acid (Yuan et al. 2016). Other compounds, such as dexrazoxane (a cardioprotective medication) and deferoxamine (an iron chelator), have also demonstrated the ability to suppress ferroptosis. The secondary functions of iron chelation therapy remain secondary to the primary goal of decreasing iron accumulation in the body.

**TABLE 2:** Clinical trial status of novel inhibitors of ferroptosis.

Drug	NCT	Phase	Intervention	Status
M 30	NCT02409030	Observational	Alzheimer	Completed
M 30	NCT05627362	Phase 2	Primary Sclerosing Cholangitis	Recruiting
M 30	NCT05798169		Sarcopenia	Recruiting
M 30	NCT00985868	Phase 1	Solid Tumor	Completed
M 30	NCT01791595	Phase 1	Solid Tumor	Completed
M 30	NCT01188252	Phase 1	Neoplasms	Completed
Baicalein	NCT03830684	Phase 2	Influenza	Not recruiting
Rosiglitazone	NCT04114136	Phase 2	Solid Malignant Tumor	Recruiting
Rosiglitazone	NCT00405015	Phase 2	Ischemia	Completed
Pioglitazone	NCT00982202	Phase 2	Alzheimer Disease	Completed
Pioglitazone	NCT01931566	Phase 3	Alzheimer's Disease	Terminated
Pioglitazone	NCT02284906	Phase 3	Alzheimer's Disease	Terminated
Pioglitazone	NCT01280123	Phase 2	Parkinson's Disease	Completed
Troglitazone	NCT00758719	Observational	Degenerative Disc Disease	Completed
Troglitazone	NCT00003058	Phase 2	Sarcoma	Completed
Zileuton	NCT02047149	Phase 1	Chronic Myelogenous Leukemia	Terminated
Zileuton	NCT00056004	Phase 2	Head and Neck Cancer	Completed
Zileuton	NCT04996199	Phase 4	Trigeminal Neuralgia	Recruiting
Dexrazoxane	NCT01627938	Phase 2	Multiple Sclerosis	Unknown
Dexrazoxane	NCT00544778	Phase 2	Sarcoma	Terminated
Dexrazoxane	NCT00742924	Phase 1	Sarcoma	Completed
Deferoxamine	NCT04566991	Phase 2	Aneurysmal Subarachnoid Hemorrhage	Recruiting
Deferoxamine	NCT00777140	Phase 1	Ischemic Stroke	Completed

### 4.1 Small Molecule Inhibitors Targeting the Iron Reduction

A direct approach to inhibiting ferroptosis involves reducing the levels of iron ions. One such inhibitor is the iron chelator ciclopirox olamine (CPX), known for its broad‐spectrum antifungal and antibacterial properties (Dixon et al. 2012). The olamine salt form of CPX (CPX‐O) has been shown to cause the destruction of ferritin and prevent ferroptosis by chelating iron ions in animal models of polycystic kidney disease (Radadiya et al. 2021). Another commonly used iron chelator is deferoxamine, which has been explored for its potential to treat traumatic spinal cord injury by inhibiting ferroptosis (Dixon et al. 2012). Additional iron chelators like deferiprone (DFP) and deferasirox (DFX) have also been reported, expanding the options beyond CPX and deferoxamine (Zheng et al. 2021). These compounds do not strictly meet the definition of ferroptosis inhibitors, yet their iron‐chelating ability indirectly suppresses ferroptosis activity.

### 4.2 Small Molecule Inhibitors Targeting Lipid Peroxide Reduction

Ferrostatin‐1 was proposed as a potent inhibitor of erastin or RSL3 in HT‐1080 cells‐induced ferroptosis. The primary aromatic amine in Fer‐1 has been shown to inhibit lipid peroxidation (Dixon et al. 2012) and may prevent ferroptosis development triggered by an overactive p53. Studies have demonstrated that Fer‐1 can elevate GSH levels to prevent ferroptosis caused by RSL3 or erastin in oligodendrocytes (Skouta et al. 2014; Weiland et al. 2019; Wu et al. 2018). Liproxstatin‐1 is another lipid peroxidation inhibitor that is frequently used in the management of ferroptosis. Fer‐1 has comparable inhibitory characteristics, including the ability to prevent erastin‐induced ferroptosis. Furthermore, in the absence of GPX4, liproxstatin‐1 can stop cells from going through ferroptosis, providing a protective factor in various disease models (Friedmann Angeli et al. 2014).

### 4.3 Small Molecule Inhibitors Modulating the GSH/GPX4 Axis

The inhibition of System Xc, responsible for cellular cystine uptake, is associated with preventing ferroptosis, and one such inhibitor is β‐mercaptoethanol (Dixon et al. 2012). In this mechanism, cystine and ‐ME form a mixed disulfide, which enters cells through System L and rapidly converts back to cysteine (Ishii et al. 1981). Increasing the expression of GPX4 can reduce ferroptosis. Selenium (Se) controls this process by activating transcription factors TFAP2c (transcription factor starting protein 2 gammas) and Sp1 (specificity protein 1), which inhibitors of transcription can prevent. However, it has been observed that Se cannot protect cells with low GPX4 levels (Alim et al. 2019)—nucleic acids and proteins with inhibitory properties.

Several proteins involved in iron metabolism play a role in regulating ferroptosis. Prominin2 promotes the production of exosomes and multivesicular bodies and regulates ferritin secretion from cells, enhancing ferroptosis resistance (Brown et al. 2019). Non‐coding RNAs, such as LINC00336, also have inhibitory effects. The long non‐coding RNA RP11‐89 prevents ferroptosis by controlling miR‐129‐5p, which has an inducing function for ferroptosis (Du and Guo 2022). The enzyme arachidonate lipoxygenase 15 (ALOX15) is targeted by miR‐522 to inhibit ferroptosis (Zhang et al. 2020). The activity of acetyl‐CoA carboxylase 1 (ACC1), which is involved in fatty acid synthesis, is regulated by the LKB1‐AMPK (liver kinase B1) axis, which also influences ferroptosis. AMPK activation leads to phosphorylation and inhibition of ACC1, a downstream substrate of AMPK (Herzig and Shaw 2018; Li, Dong, et al. 2020).

Without GPX4, M1 tumor‐associated macrophages exhibit increased resistance to ferroptosis compared to the M2 subtype, as they follow a different metabolic pathway that suppresses lipid ROS formation (Kapralov et al. 2020). Nitric oxide (NO) synthase (iNOS) is expressed by M1 subtype cells and produces NO, which can stop lipid peroxidation and ferroptosis in these cells (Xu, Ye, et al. 2021). The Wnt/β‐catenin pathway can be inhibited by Dickkopf‐1 (DKK1), which also promotes tumor growth (Igbinigie et al. 2019). Recent findings suggest that DKK1 is a ferroptosis inhibitor crucial for metastatic disease progression. DKK1 upregulates the expression of a subunit of System Xc, SLC7A11, to exert its inhibitory effect (Wu et al. 2022). DKK1 indirectly decreases the activity of STAT3 by blocking the Wnt/β‐catenin pathway, which binds to the promoter region of SLC7A11 and inhibits its expression (Fragoso et al. 2012; Linher‐Melville et al. 2015).

**FIGURE 4:**
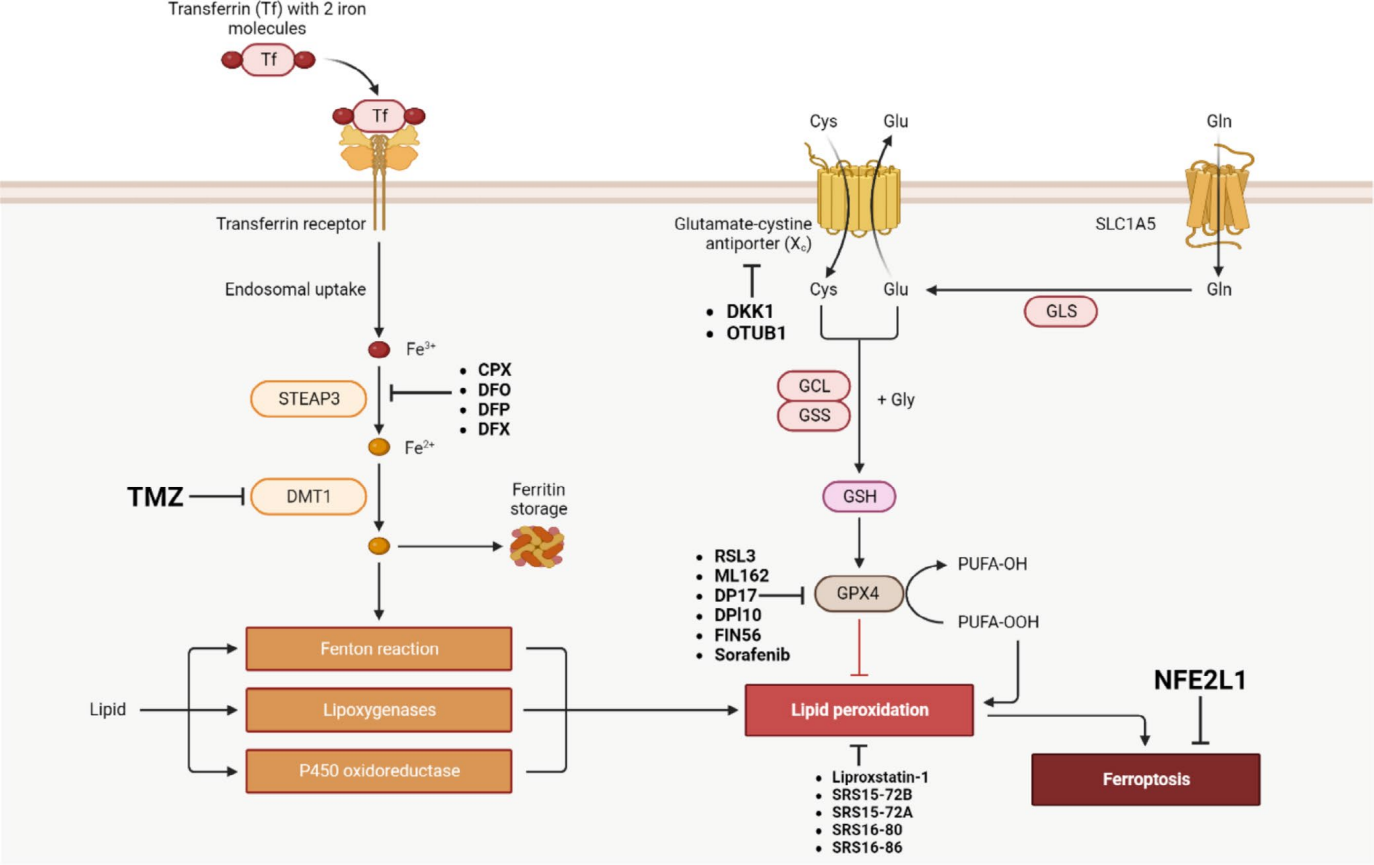
Multiple types of inhibitors are involved in blocking the ferroptosis pathway.

Additionally, it has been demonstrated that the deubiquitinase OTUB1 stabilizes SLC7A11. High amounts of OTUB1 in tumor cells prevent ferroptosis (Liu et al. 2019). The transcription factor NFE2L1 controls proteasomal activity (Bartelt and Widenmaier 2020). Cell tolerance is improved, and ferroptosis inducers increase proteasomal activity. Although the precise mechanism by which proteasomal activity prevents ferroptosis is unknown, evidence points to a possible connection with ubiquitination (Kotschi et al. 2022). A combined summary of all inhibitors involved in blocking the ferroptotic pathway is shown in Figure 4.

### 4.4 Iron‐Chelating Compounds as Potential Therapies for Iron Overload and Ferroptosis

Deferasirox, deferiprone, and deferrioxamine are the iron‐chelating medications most commonly used in clinical practice (Yan et al. 2018). While these chelators are primarily employed to treat iron overload associated with thalassemia, they may also have applications beyond addressing iron accumulation caused solely by blood transfusions (Kontoghiorghes and Kontoghiorghe 2020). The inhibitory action of iron chelators on ferroptosis occurs through reducing available iron but does not target the ferroptosis‐specific mechanisms.

Hepcidin supplementation, for instance, can lessen intestinal iron absorption (Katsarou and Pantopoulos 2018; Swinkels and Drenth 2008). Transferrin (Tf), which can be genetically created or isolated from human serum, is a natural iron‐chelating substance used to treat iron overload in particular body areas (Bobik et al. 2019). Research currently lacks experimental evidence to prove that either hepcidin or transferrin directly suppresses ferroptosis despite their indirect anti‐ferroptosis effects caused by removing iron from cells. However, these options are challenging to obtain, exhibit low yields, and possess limited activity, making them suitable for scientific research but less applicable in clinical settings. Consequently, the search for the optimum iron chelator in plant bioactives is predominately focused on flavonoids since these compounds also have antioxidant capabilities and control iron homeostasis, lower prices, and fewer side effects (Lesjak and Srai 2019).

## 5 Conclusion and Future Perspectives

The understanding of ferroptosis as a driver of cell death and its involvement in diseases like cancer is well‐established, but there is still a lack of information regarding its role in neurodegenerative diseases. Most of the available data on ferroptosis come from preclinical models, and further clinical investigations are needed to bridge the gap between iron chelators and antioxidant nutraceuticals, which have shown promise in experimental settings as potential therapeutic tools to halt the process of ferroptosis in neurodegenerative disorders. However, their efficacy in human trials has been limited, indicating the need for research focusing on signaling molecules from other pathways contributing to ferroptosis. New medication delivery strategies and improving flavonoid dissolution techniques have become crucial study topics. Utilizing nanomaterials, for example, could offer a viable strategy for enhancing flavonoids' effectiveness as iron chelators. Their concentration and the pH of the environment affect the capacity of flavonoids to chelate iron, which, in turn, affects usage and dosage circumstances. Continued research in this area bodes well for discovering novel therapeutic options for people with neurodegenerative diseases.

Future research in ferroptosis of neurological disorders requires focusing on several promising pathways to enhance our knowledge about these mechanisms. The interplay between GSH, ROS, GPX4, and ferroptosis highlights the complexity of the disease's mechanisms and offers potential targets for therapeutic interventions.

While the precise mechanism by which the mutant huntingtin protein causes neurodegeneration remains elusive, it has been demonstrated to induce oxidative damage. Notably, experiments using yellow fluorescent protein labeled neurons revealed that fer‐1 treatment at various dosages (10 nM, 100 nM, and 1 M) conferred protection against cellular damage. Conversely, biolistic transfection of a huntingtin exon 1 segment containing the pathogenic repeat (73Q) (mN90Q73) resulted in cell death (Skouta et al. 2014). Furthermore, administering the iron chelator deferoxamine has shown preventive effects in R6/2 mice (Yang et al. 2016).

**FIGURE 5:**
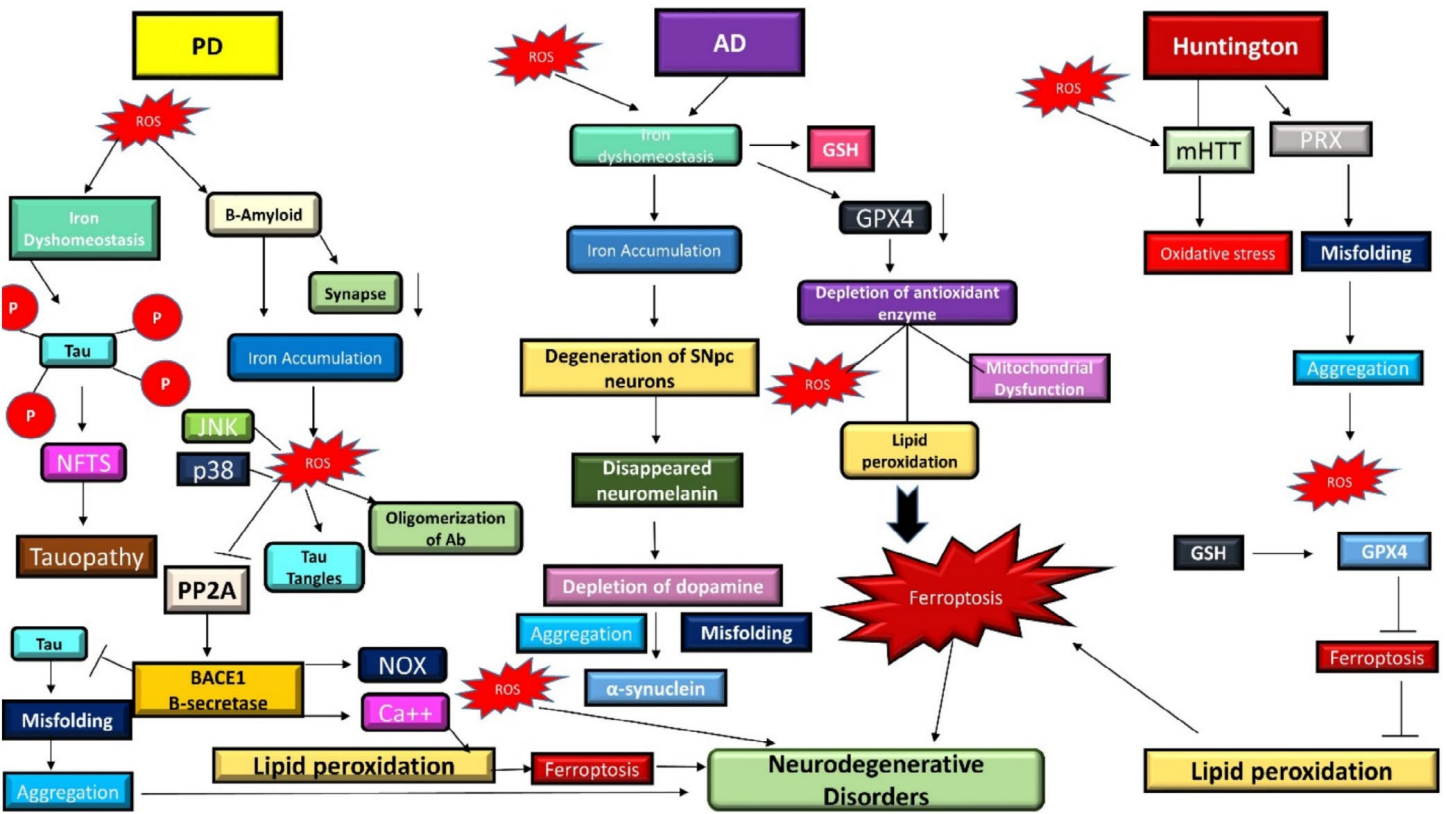
The interplay of ferroptosis in Alzheimer's disease (AD), Parkinson's disease (PD), and Huntington's disease (HD).

To understand the role of ferroptosis in the nervous system and its significance in neurological disorders, developing suitable chemical probes or biomarkers is crucial, given the challenges of obtaining sufficient neurons for study (Figure 5) (Guiney et al. 2020). Potential indicators of ferroptosis in the neurological system include mitochondrial shrinkage and immune‐related cells and factors, setting it apart from other cell death pathways (Ren et al. 2020).

Recent advancements in nanoparticle technology development offer promising avenues for studying ferroptosis in the brain. Nanoparticles with flexible characteristics such as size, charge, and targeting ligands exhibit high sensitivity in the brain, making them potential models for the development of novel anti‐ferroptotic nanomaterials in the future (Xu et al. 2020). Additionally, it is crucial to consider ferroptosis as a possible adaptive modification in the body's response to various stimuli (Dixon 2017).

The interaction between redox phosphatidyl liposomes, which include polyunsaturated fatty acids (PUFA) and phosphatidylethanolamine (PE), and catalytic or regulatory proteins such as GPX4, PEBP1, and 15‐LOX, can offer valuable insights into the critical pathogenesis of ferroptosis and serve as potential targets for pharmacological interventions (Wenzel et al. 2017). Finally, when evaluating the impact of different ferroptosis inhibitors, considering their combination with other cell death inhibitors and exploring alternative medication delivery systems can enhance therapeutic outcomes. These considerations contribute to a comprehensive understanding of the role of ferroptosis in neurological disorders and pave the way for potential therapeutic interventions.

## Author Contributions

**Felix Kwashie Madilo:** data curation (equal), resources (equal), writing – review and editing (equal). **Anwar Ali:** conceptualization (equal), resources (equal), writing – original draft (equal). **Quratulain Babar:** conceptualization (equal), methodology (equal), supervision (equal). **Ayesha Saeed:** formal analysis (equal), investigation (equal), writing – review and editing (equal). **Domenico Sergi:** methodology (equal), supervision (equal), writing – review and editing (equal). **Isam A. Mohamed Ahmed:** conceptualization (equal), funding acquisition (equal), writing – original draft (equal). **Ghalia Shamlan:** funding acquisition (equal), visualization (equal), writing – review and editing (equal). **Halah Abdulrahman Hafiz:** project administration (equal), visualization (equal), writing – original draft (equal). **Nenad Naumovski:** conceptualization (equal), validation (equal), writing – original draft (equal). **Muhammad Faisal Manzoor:** funding acquisition (equal), supervision (equal), writing – review and editing (equal). **Joanna Trafialek:** funding acquisition (equal), validation (equal), writing – review and editing (equal).

## Conflicts of Interest

The authors declare no conflicts of interest.

## Data Availability

Data sharing not applicable—no new data has been generated for this research.
